# Supplementary figures and images for: The domesticated transposase ALP2 mediates formation of a novel Polycomb protein complex by direct interaction with MSI1, a core subunit of Polycomb Repressive Complex 2 (PRC2)
Source: PLoS Genet. 2020 May 28;16(5):e1008681. doi: 10.1371/journal.pgen.1008681 (PMC7282668; doi:10.1371/journal.pgen.1008681)

***SEP3***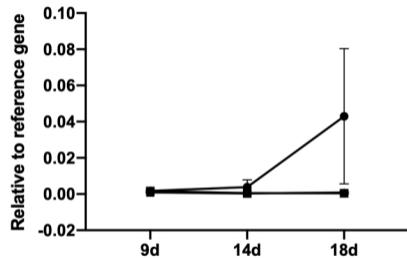***AG***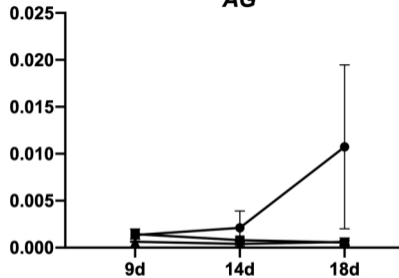***PI***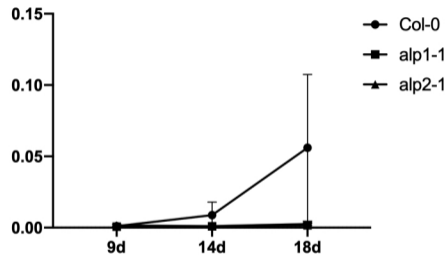

● Col-0  
■ alp1-1  
▲ alp2-1

Days post LD shift

Supplement: S1 Fig — RT-qPCR of SEP3, AG, and PI in SAM-enriched tissue of Col-0, alp1-1, and alp2-1 plants that were grown under short day conditions (8 hr light, 16 hr dark) for 14 days, and then grown under long day conditions (16 hr light, 8 hr dark). Tissue was collected on the days specified after the photoperiod shift 1 hr prior to the commencement of the dark period (ZT 15). Values are the mean of three biological replicates and are presented relative to the reference gene PP2AA3 (AT1G13320). Error bars indicate the standard deviation of the mean (PDF) [file pgen.1008681.s001.pdf]

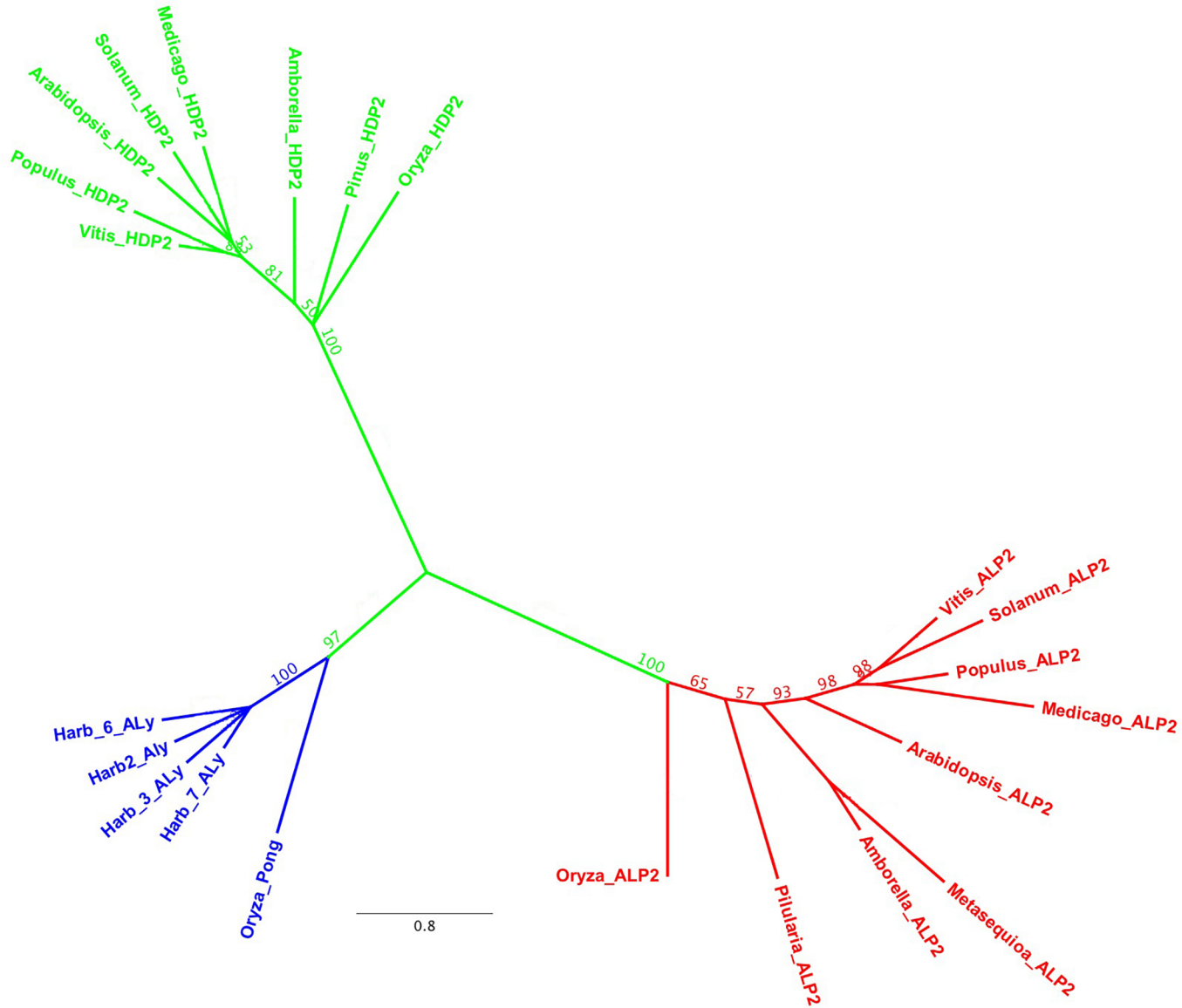

Supplement: S2 Fig — An unrooted Bayesian phylogenetic tree was made based on alignment of the amino acid sequences. Numbers at the branches indicate probabilities (percent), the scale bar indicates the average number of substitutions per site. ALP2 sequences are indicated in red, Harbinger transposases in blue and HDP2 sequences in green. (PDF) [file pgen.1008681.s002.pdf]

*A. lyrata*

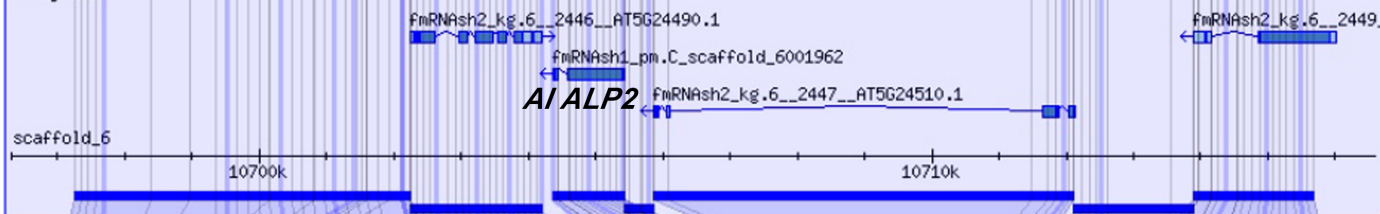

*A. thaliana* (reference)

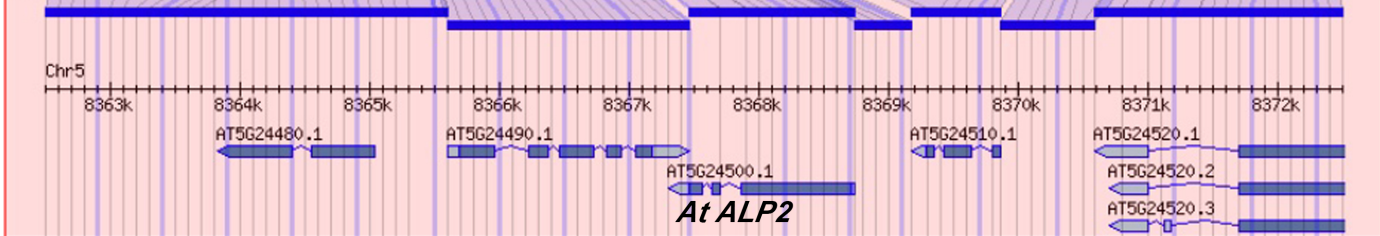

Supplement: S3 Fig — In A. thaliana, ALP2 is neighboured by At5G24490 and At5G24510 encoding 30S and 60S ribosomal proteins, respectively. The A. lyrata ALP2 orthologue (Al ALP2) is located in a syntenous region, i.e. is flanked by orthologous genes with the same arrangement and orientation as in A. thaliana. (PDF) [file pgen.1008681.s003.pdf]

35S::GFP

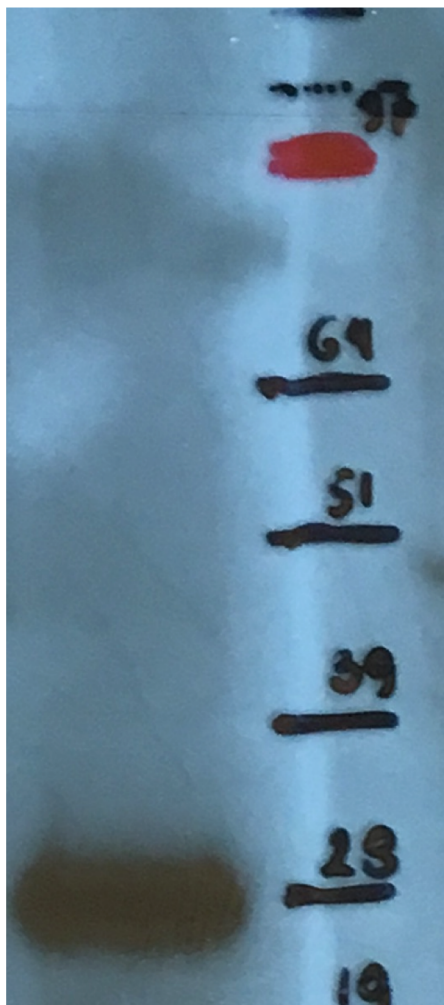

pLHP1::LHP1-GFP

35S::GFP-ALP2

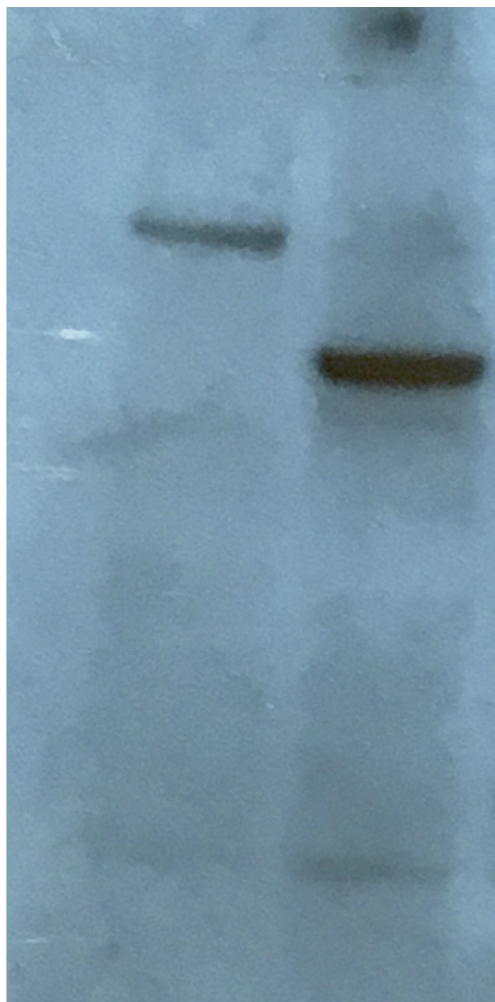

Supplement: S4 Fig — Immunoblot analysis of total protein extracts using α-GFP antibodies. A protein with the predicted size (65.2 Kda) for the ALP2-GFP fusion is specifically detected in extracts from 35S::GFP-ALP2 plants. 35S::LHP1-GFP is included as a positive control. (PDF) [file pgen.1008681.s004.pdf]

# ALP2\_vs\_Ctrl

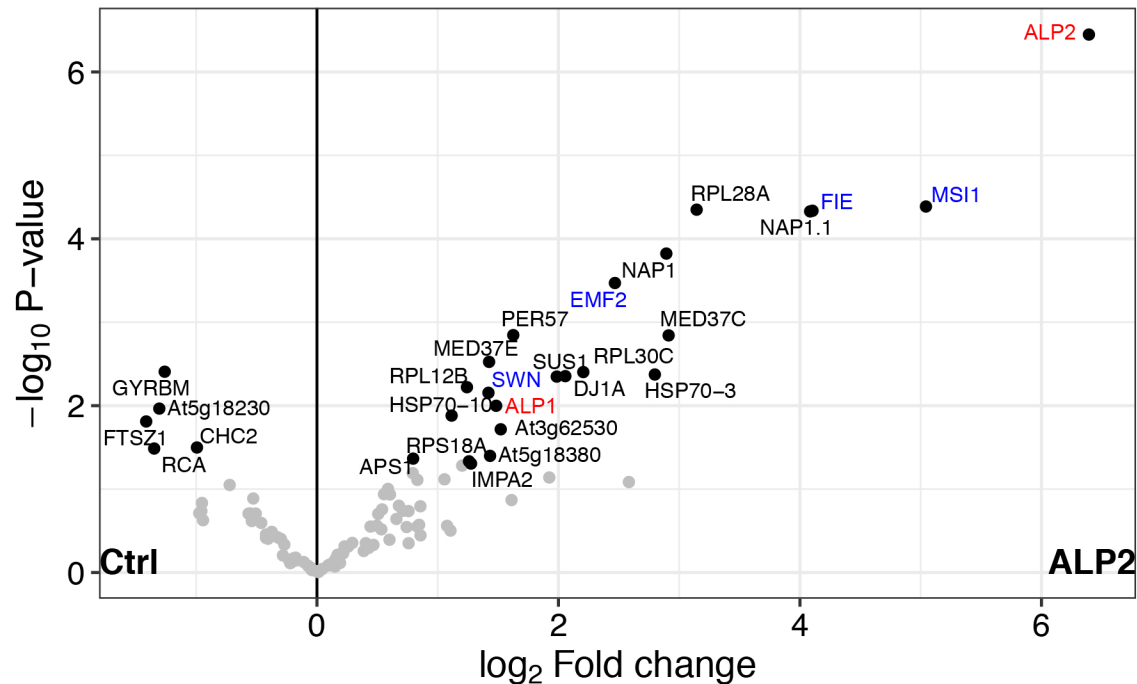

Supplement: S5 Fig — Volcano plot analysis with the x axis showing relative abundance of proteins identified in tandem affintity purified 35S:GSrhino-ALP2 samples relative to non transgenic control. The y axis shows probability values. Analysis is based on three biological replicates. ALP proteins highlighted in red, PRC2 components in blue. (PDF) [file pgen.1008681.s005.pdf]

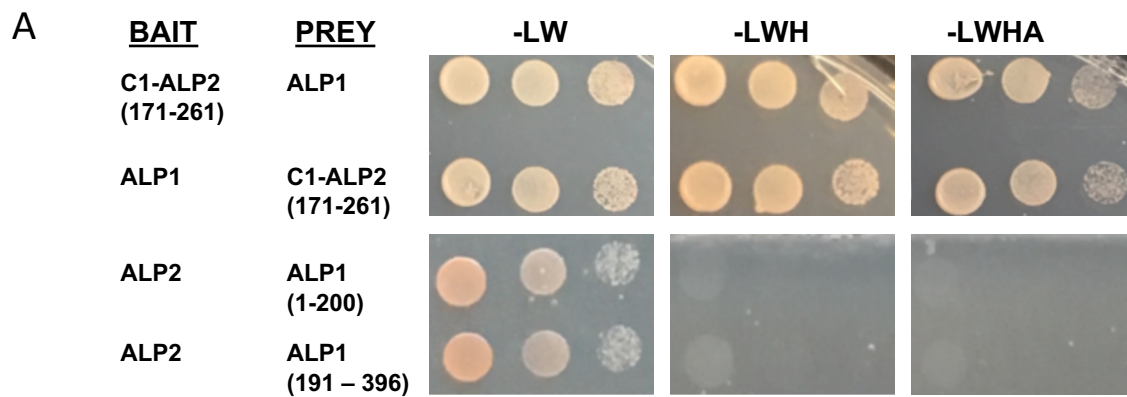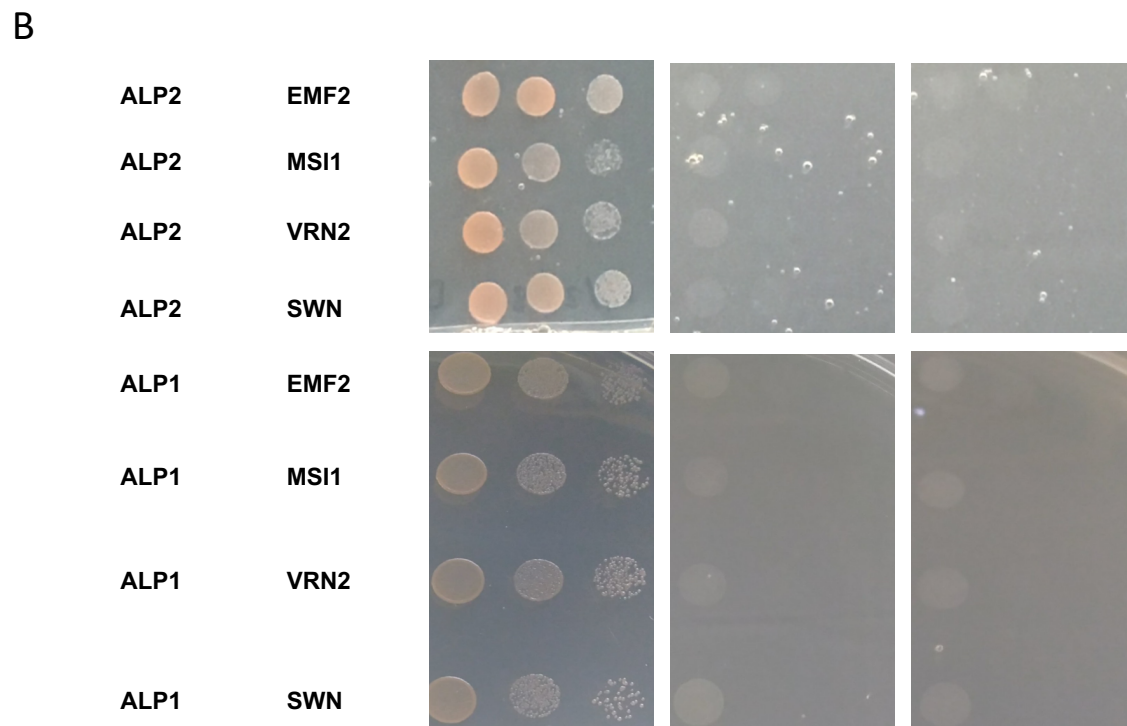

Supplement: S6 Fig — A. Truncated forms of ALP1 did not interact with ALP2. The interaction of a C-terminal region of ALP2 (residues 171–261) is included as a control. B. Full length ALP1 and ALP2 proteins did not interact with core PRC2 components. (PDF) [file pgen.1008681.s006.pdf]

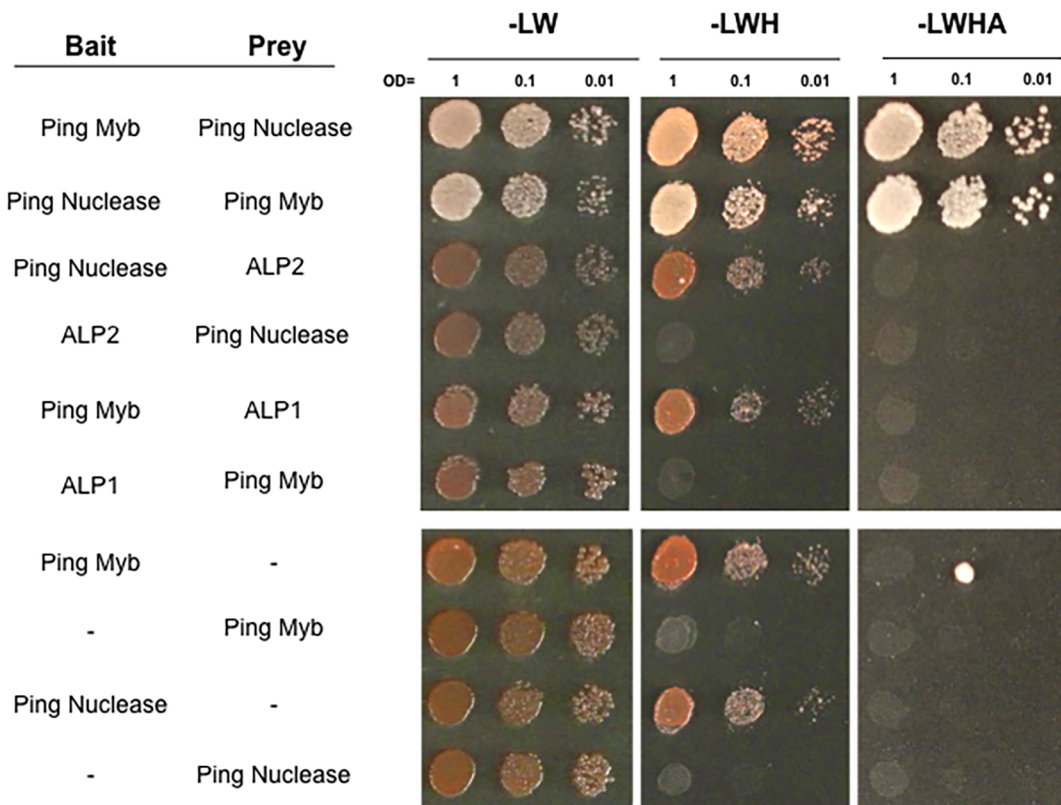

Supplement: S8 Fig — The full length Ping nuclease and Myb DNA binding proteins interact reciprocally as bait and prey fusions. No interaction above background was found between ALP and Ping proteins. Serial ten-fold dilutions of five pooled transformants were spotted onto selective media. (PDF) [file pgen.1008681.s008.pdf]
