# Supplementary material for: The domesticated transposase ALP2 mediates formation of a novel Polycomb protein complex by direct interaction with MSI1, a core subunit of Polycomb Repressive Complex 2 (PRC2)
Source: PLoS Genet. 2020 May 28;16(5):e1008681. doi: 10.1371/journal.pgen.1008681 (PMC7282668; doi:10.1371/journal.pgen.1008681)
Supplement: S7 Fig — Low magnification images showing epidermal cells from N. benthamiana leaves transformed by infiltration with Agrobacterium. Images in left of panels are YFP channel, on right is merge of light field and fluorescence channels. ALP proteins are tested with each other and with the core PRC2 components. Interactions were only seen for the ALP1-ALP2 and MSI1-ALP2 combinations. (PDF) [file pgen.1008681.s007.pdf]

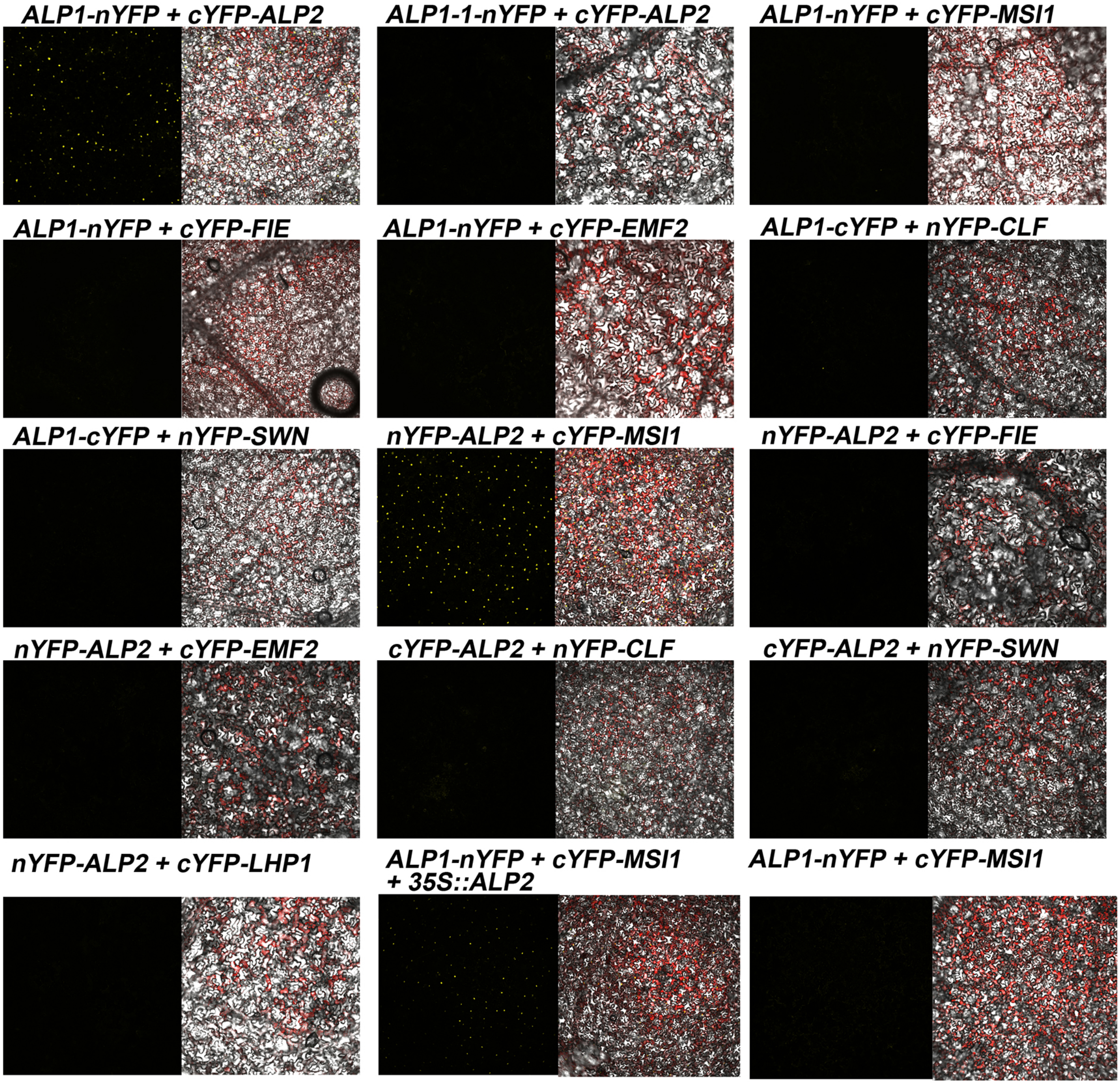
ALP1-nYFP + cYFP-ALP2
ALP1-1-nYFP + cYFP-ALP2
ALP1-nYFP + cYFP-MSI1
ALP1-nYFP + cYFP-FIE
ALP1-nYFP + cYFP-EMF2
ALP1-cYFP + nYFP-CLF
ALP1-cYFP + nYFP-SWN
nYFP-ALP2 + cYFP-MSI1
nYFP-ALP2 + cYFP-FIE
nYFP-ALP2 + cYFP-EMF2
cYFP-ALP2 + nYFP-CLF
cYFP-ALP2 + nYFP-SWN
nYFP-ALP2 + cYFP-LHP1
ALP1-nYFP + cYFP-MSI1 + 35S::ALP2
ALP1-nYFP + cYFP-MSI1
