# Supplementary material for: The domesticated transposase ALP2 mediates formation of a novel Polycomb protein complex by direct interaction with MSI1, a core subunit of Polycomb Repressive Complex 2 (PRC2)
Source: PLoS Genet. 2020 May 28;16(5):e1008681. doi: 10.1371/journal.pgen.1008681 (PMC7282668; doi:10.1371/journal.pgen.1008681)
Supplement: S9 Fig — A N-terminal fragment (amino acids 1–223) of the Ping nuclease interacts with the full length Ping Myb DNA binding protein reciprocally. A C-terminal fragment (amino acids 292–465) of the Ping Myb DNA binding protein interacts with the Ping nuclease as a bait but not as a prey fusion. No interaction was found between truncated ALP proteins and Ping proteins. Serial ten-fold dilutions of five pooled transformants were spotted onto selective media. (PDF) [file pgen.1008681.s009.pdf]

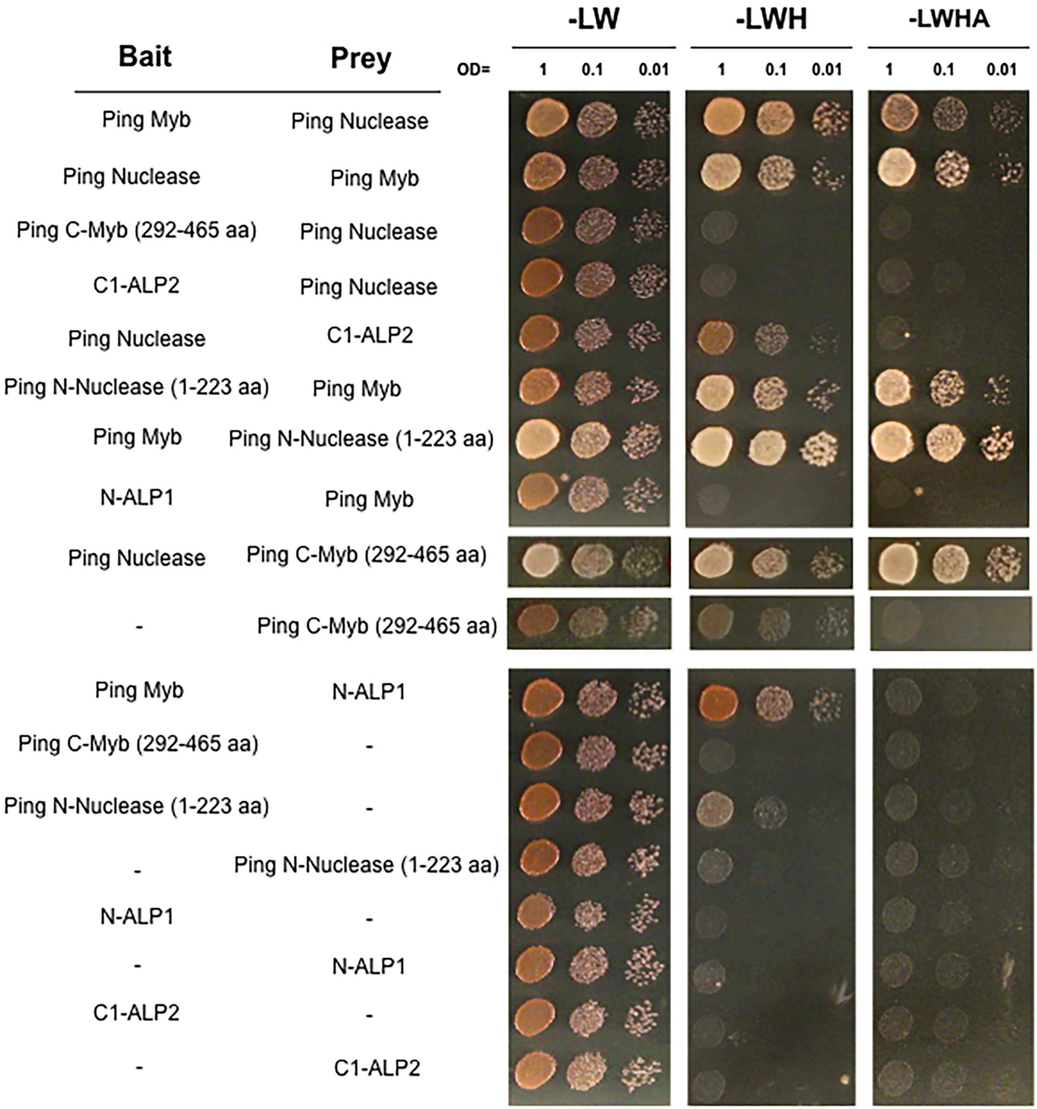

Bait
Prey
-LW
-LWH
-LWHA
OD=
1
0.1
0.01
Ping Myb
Ping Nuclease
Ping Nuclease
Ping Myb
Ping C-Myb (292-465 aa)
Ping Nuclease
C1-ALP2
Ping Nuclease
Ping Nuclease
C1-ALP2
Ping N-Nuclease (1-223 aa)
Ping Myb
Ping Myb
Ping N-Nuclease (1-223 aa)
N-ALP1
Ping Myb
Ping Nuclease
Ping C-Myb (292-465 aa)
-
Ping C-Myb (292-465 aa)
Ping Myb
N-ALP1
Ping C-Myb (292-465 aa)
-
Ping N-Nuclease (1-223 aa)
-
-
Ping N-Nuclease (1-223 aa)
N-ALP1
-
-
N-ALP1
C1-ALP2
-
-
C1-ALP2
