# Supplementary material for: The domesticated transposase ALP2 mediates formation of a novel Polycomb protein complex by direct interaction with MSI1, a core subunit of Polycomb Repressive Complex 2 (PRC2)
Source: PLoS Genet. 2020 May 28;16(5):e1008681. doi: 10.1371/journal.pgen.1008681 (PMC7282668; doi:10.1371/journal.pgen.1008681)
Supplement: S1 Table — A. Annotation of high scoring mutations from fast isogenic mapping identified two candidate genes (Hartwig et al. 2012). Score indicates Shore quality score ranged from 1 (low) to 40 (high). Protein position indicates amino acid number in translated coding sequence. B. Allele frequency at the two candidate mutations reveals high frequency of the mutant allele in DNA from bulked mutant plants. (PDF) [file pgen.1008681.s010.pdf]

A

Chromosome	Position	Mutation	No reads	Concordance	Score	Annotation	Gene	Protein position	Type	Amino acid change
**5**	**8368077**	**C->T**	**51**	**0.96**	**40**	**CDS**	**AT5G24500**	**213**	**Nonsyn**	**W->***
**5**	**8774239**	**C->T**	**50**	**0.98**	**40**	**CDS**	**AT5G25280**	**120**	**Nonsyn**	**P->S**
5	10761168	C->T	50	0.86	36	intergenic	-	-	-	-
5	14126583	C->T	31	0.84	36	intergenic	-	-	-	-

B

Chromosome	Position	SNP	Coverage	Ref C (%)	SNP T (%)	other (%)
5	8368077	T	12702	1.48	98.52	0
5	8774239	T	61078	3.193	96.804	0.003

S1 Table. Identification of *ALP2* candidate genes by sequencing. A. Annotation of high scoring mutations from fast isogenic mapping identified two candidate genes (Hartwig et al. 2012). Score indicates Shore quality score ranged from 1 (low) to 40 (high). Protein position indicates amino acid number in translated coding sequence. B. Allele frequency at the two candidate mutations reveals high frequency of the mutant allele in DNA from bulked mutant plants.
