# Supplementary material for: The domesticated transposase ALP2 mediates formation of a novel Polycomb protein complex by direct interaction with MSI1, a core subunit of Polycomb Repressive Complex 2 (PRC2)
Source: PLoS Genet. 2020 May 28;16(5):e1008681. doi: 10.1371/journal.pgen.1008681 (PMC7282668; doi:10.1371/journal.pgen.1008681)
Supplement: S2 Table — Table shows the number of uniquely identified peptides from ALP proteins, core PRC2 subunits and accessory components. Three replicate experiments. Non transgenic PSB-D Arabidopsis suspension culture cells were used as control. (PDF) [file pgen.1008681.s011.pdf]

Protein	PSB-D control cells	GS^{rhino}-ALP2 cells
ALP2	0-0-0	10-7-7
ALP1	0-0-0	7-4-2
EMF2	0-0-0	8-7-7
SWN	0-0-0	8-3-0
FIE	0-0-0	13-10-4
MSI1	0-0-0	11-7-10
CLF	0-0-0	0-0-0
EMF1	0-0-0	0-0-0
LHP1	0-0-0	0-0-0
VRN5	0-0-0	0-0-0
VEL1	0-0-0	0-0-0
ALL PEPTIDES	469-517-987	2728-776-992

S2 Table. IP-MS results using *35S::GS^{rhino}-ALP2* in suspension cells. Table shows the number of uniquely identified peptides from ALP proteins, core PRC2 subunits and accessory components. Three replicate experiments. Non-transgenic PSB-D Arabidopsis suspension culture cells were used as control.
