# Supplementary material for: The domesticated transposase ALP2 mediates formation of a novel Polycomb protein complex by direct interaction with MSI1, a core subunit of Polycomb Repressive Complex 2 (PRC2)
Source: PLoS Genet. 2020 May 28;16(5):e1008681. doi: 10.1371/journal.pgen.1008681 (PMC7282668; doi:10.1371/journal.pgen.1008681)
Supplement: S1 Text — Text file with the species names, accession numbers and protein sequences used for the alignment in Fig 2 and the phylogenetic tree in S2 Fig. Sequences were mostly retrieved from Genbank, other than where indicated Harbinger transposase sequences which were retrieved from Repbase and the gymnosperm and pteridophyte sequences which were retrieved from OneKP. (DOCX) [file pgen.1008681.s013.docx]

>Amborella trichopoda ALP2 XP_011621984 MQMHCPTIPGAHNWLDKLRSSKGFPLDNGLNLENFLKSSSPSPNPNFSGDVSPPLLCHSPENSLNLINASGFSATKRDPGAWFDIMTDVLSDLFNMEGSYDFRRLRDSRDRKNGRKQSRPRICALSAEELPNFSARNNADNNSGVGGGSRDDADVDISSCSRTEVTVIDTSIATWKSEKVIYRKAHVWKVRERKGNSCGKGRGGESLVLKKRRLGQNKKGQDGVGISASTECLDDQDRRFQRRQICLASPGRKGRATGLRGRQKDS

>Amborella trichopoda HDP2 XP_006850986 MEEPAEHTPTNRREAHQKKIESDSIQTISRYTRSRAAPEWTLTEMLILVREVCSAEEECLGLSVYQKWKIVSENCGVLDVMRSANQCRRQWEMMLSDYKKIKEWELQSGIGSFWKVNVDQMKEVVGVLFLFDQELYDLINELLTTREAGPNSRADSGSEGLINLADDDGDNEFNSVLTAPKALNKAVRVRSTKKPKTTKPPPQARKIDKEHEIAEKLHENSQAIQEILQRSLAEDPCEKFTEAEINLKRQQADELVKAFGDLLGSLNQFVELVEQCD

>Arabidopsis thaliana ALP2 NP_197838.2 MLSSIIDDKPASSTWLNRLRLNRGLTTDDDDASGNPLTLDDFLRRNHHTEIAATSSASDSPPSAPIPSDPELAESPSEEPVPGEWYGVMSDVLFELFNFSGSSKSSTIPGKKKLPRKQSNPRHCSLETPEDVVVPLVNQKSDDANCLPSVREFATSSSRSSYNKKPPAPEIRERRRSVVEGDGVDEEEEKGEKDLVGFSRSEVTVIDTSFKIWKSEKLVFRRRNVWKVREKKGKSRVVSKLKKLMKKKKKKKRKCDDVDDDDGGIARKKSKKMKISTSVSDNNPRYNVEEIHDEPESSNVSRRLLSKPRKEGSFGIHTSKKNSEAAAQGYRSLA

>Arabidopsis thaliana HDP2 NP_194855.2 MEEGTSGSRRTRSQVAPEWAVKDCLVLVNEIAAVEADCSNALSSFQKWTMITENCNALDVSRNLNQCRRKWDSLMSDYNQIKKWESQYRGTGRSYWSLSSDKRKLLNLPGDIDIELFEAINAVVMIQDEKAGTESDSDPEAQDVVDLSAELAFVGSKRSRQRTMVMKETKKEEPRTSRVQVNTREKPITTKATHQNKTMGEKKPVEDMSTDEEEDETMNIEEDVEVMEAKLSYKIDLIHAIVGRNLAKDNETKDGVSMDDKLKSVRQQGDELIGCLSEIVSTLNRLHEVPQEIE

>Arabidopsis lyrata Harbinger Repbase HARB-2_Aly MDDMNPFYHSSGFMNLLQSQQEDMSQFASGSTEVPAFFTSSQLSEEGSEEEGNEASDVKPKQSISRKKWTAKEDIVLVSAWLNTSKDPVIFTGNDQQGQSFWKRIAAYVAASPSLDGMPKREHAQCKQRWAKVNKIVTKFVGCFTHKTATTHKTSGQSEDDVMKLAHEIYYNDTKKNFTLDHAWRELKFDQKWCDQFTTTRGGKDNAKRRKCGDGNASSQPINVEDDSVMTRPPGVKAAKAKGRKSATVFTNEGKKPATGKGEAGQSVEHFQNLWELKEKDWDRKEKQSKYQMLESLLSRTEFTPLSDIDLFLKNKLITELWS

>Arabidopsis lyrata Harbinger Repbase Harb-3_Aly MFDMDSRNPYTNPSSFLNLLSSQQDNPMPPPNQYAGFFTSFSAPWIDPNQAPTPAEEPVADRKSRRKWSPTEDVILISAWLNTSKDAVVSFTNEQKATAFWQRIADYFAASPKLRGLQKRAPLRCKQRWAKINEGVCKFVGSYFTEAATKQQTSGQNENDVVNLAHQIFYNDHKAKFTLEHAWRELRHDQKWCGSSFTTSKDSGSAKRKRFEXGSAQASCSMPVNIDEEEPRPAGVKAAKATAKAKAKTFTTXANWRRRQDSEGVTLYLGD

>Arabidopsis lyrata Harbinger Repbase Harb-6_Aly MNPYTNSAGFMEMLTSQQERGSLNPNACESASPSIRLFTGESDLPAYSTQWTEATEATTQGGSAKAKSTPRKRNKWTSQEDLVLISAWLNFTTSKDAIVGNEQRGVAFWKRIAAYYAASPNISGEKKREPTHFKQRWAKINEQFTVCKFVGSFQAAAGQVSSGMNDDDLIKLAHQIFEADYDNKFNLEHAWRALRNFTEQKWCQEQSTKSTDKNKRRKVDSEASQAHNESQASQSRPAGVKAAKAKLKKFTPATTAPLVEEEGQAVERIQALWEIRQKEFALKQQEHAFRKELTNSKLLXSLFTLSKSDPLSEIEEELKNKLIRDILL

>Arabidopsis lyrata Harbinger Repbase Harb-7_Aly MDSYPYSHGSGFVNLLTSQQEVHNLEANPYDDVPVFPFTSQADSPSQAAHKPKVKRGRRKWSPTEDVVLISAWLNTSKDPVIGNEQKGNAFTFWSRIANYYGASPNLKGVERREPDHIKQRWAKINDGVCKFVGSYEAALKQQFTSSGQNDDDVMKLAHQIFTNDYDAKFNLEHAWRELRHDQKWCSAPTTKPTGQFTSKRRKTKESVEPSSCQPLSHGVEEAMHRPIGVKAAKANAKKTVGEEGNGLSFTRLQGIWEISKKNFELKEKQSKHSMLDSLLAKTELSDTEIALKDKLINDMLSFTNA

>Medicago truncatula ALP2 XP_003602376 MICSPRSNKPGSNWLDRLRSNKGIPTDDNLDLDTFILNLATHSPQPRPIKPLRHRPPITHDDPPLTTVLAHLFNPGAATITSKKCPRKQTNPKIFIPSSTIISTTTANAPAATGVDAAVDVENRGVEGEDGEEDFKGFTKSEVTVIDTSCPVWKVDKFVFRRNNVWKIRERKQKNKFVAKKKSKSTHELDIHGIGSSKDNTINTGGEKPVKKLKVI

>Medicago truncatula_ HDP2 XP_003588618 MTENTRDAVGSRRTRSQAAPEWTLTESLILLNEIAAVEADCSVNFSSYQQWDIISQNCAALDVDRNLAQCRRKWRSLLAEYEEVRRRRRAWNLDRELFDAVERVVKGREERGEVDRESDGELGNEERDVTVEIDMIGTGFKRKRPRSKVERNSVQKPKKYWPVEPSENLKEKQVPEEIHEEVPENNHMEEEFLKDFLEKKPKLKSPAKRRQKHIETPLQNLPEVDKHHDIEKPKPASVGNITNNSREENEEMLTLKLQELAIEIQEISAESADCKEANTENIEDYRTEFTRRQGDKLIARLGNFSNTLKQLCDLLQECK

>Metasequoia_glyptostroboides OneKP NRXL-2008643 EARMNWLNRLRSAKGFPVEPGLRMEDFLNSTTSQDRSPALSCTKSCQVKTENGEAQGDEKGRARVKGATKKKSLAAVKEEKKDWLEMTNNALAELFCMRQDWKLDDMKKTKRKRKQDKPRICPSLDANSLSPLSANDDNIVERPGKASDKAVSTSSPLSAVNSVVAGAGKKEYDGEISPVKKRKGSRQLVEDSIDGSKRSNADISSSSCNSCTEVTLIDTSLSTWRSRKVMYKKGSIWKVGEKKAQSVS

>Oryza sativa ALP2 XP_015643506.1. MPCCSAVLSAAAAAAAASRTPPWLHRLHAKGGLSFPSNLHIDDLLYGQHHALPHPPPPPPPPQPAKEPPPPTKPKHPKPKQQQHPPPPPPQKPPQGSTNLSLPNPSGSGSGNGNPPPSPQLQLSTVIADVFVTPSSAPPLIAPIKAFRKQNHPRPRPDKASRPSKENKDKASKVKVKKRRRSDRAADGDGERCSRTEVTVIDTSTDGWKAAKLLLRRGAVWKVRDKASGVSEPEDPTKMKRRAGLVSKIQRDREKQKQKEKEATSSGNIHASSGDGMKEPDGPIQALKRSRGPEPEPEII

>Oryza sativa HDP2 XP_015626063 MAMDPDAAEWPCIIQALPALPPSPSSTGVPRLPTMVQALPAATDPPPAAARLRRGAEPPSPRRTRSGGAPEWTPAETLALVAEVAAVDDGWSRSVSAFQKWAIVAENLAASRGRAARGRGRGRAASECRRRWEALAAEYGVVRRWEVRGAGGYWRMSAAARRKAGLPADFDAEVYGAMEALTLVEEALLADATAGAGGAEIGEKSAAAAEVGEGDEVEAGEEDGNGDRGEVGEEDEGEVGEDGEEEDGNEMVEVEDDGNADDKDAQPDGGNAAASDDLEVCETGANNEGKKSQTDACELANKLQENAQHIHMLLKEEAGENENHNLAISSDSMETTRQKGDELIKSLGGLVSYLNQFTDLIKENGFENVVGMS

>Oryza_sativa Pong DAA00397.1 MFDFLMLPSVSIGFASYVFRLYPTEGFVNFLQQNCLPQPQEGENFHLVGQTTNTMSTPPPTPQAAANNTVQIDIHEDAINDASAKKRSLRYWTHDEEERLASAWLNASKDPIHGNEKKGDTFWKEVTDEFNRKGNGKRTREINQLKVHWSRLKSSIGEFNDYWTKVTQMNTSGYDDDMLEKEAQQMYANTFGKPFALVHWWKILRKEPKWCAMIEKDKNKAEVVDIPDEQKRPIGREAAQAERNGKRKKDSMSEGIVILGDNIEKIIKVTQDRKLEREKVTEAQIHISNVNLKAAEQQKEAKMFEVYNSLLTQDTSNMSEEQKARRDKALQKLEEKLFAD

>Pilularia globulifera ALP2 OneKP KIIX-2008112 DMLSELFCMESCSSNSEAMKKRPRKQGNPKNCNIFPDLEPSCTAITSVDVPMEESNKNSSREIVSENPPTLLSAAATLEKVDIRSSSQGAMAADYALCQQKSSKKESVGFKRRKYNCDNDVILENQVYLIDTSVPGWKVEKLITRRGNSWKVQNKEASISREGSLGKPMKVPKRRARTMED

>Populus trichocarpa ALP2 XP_002318455 MLCSVQTSKSSSNWLDRLWSNRGFNNNNDNNPSVPNPSSSPTTNASNSVINSNSESTHSDSDQIKVTATTATATTREISSSDNKDLFFIMNNVLSDLFNMGGVSDPVEESSRLSRKKEKVPRKQTKPKFCFISGNNSGNDSLDCVRKDRNVLAATGSLNSDKNSNNVDCGVVVDDDDDDEEDVEEDVEEEKGFGVGGDKELKGYSRSEVTVIDTSCQVWKFDKLVFRKKNVWKVRDKKGKSWVFGSKKRKGNDLESANGNGAKKKAKVSNLEVGSSKDVNDVQKQEDERREEEHKQMPEDLSQVPKKRFHFSRSPEKSIKSGSSVILIKTIPTSNKSGKNITKNRLKDNQRKNKT

>Populus_trichocarpa HDP2 XP_006371828 MADIEMEATQSITAINNKKRKKGNQDCKNSNSSRVLSTPRTRSQVSPEWTAKQALILVNEIAAVEKDCSKAVSTNQKWKIIVGNCVALGVTHPLSQCRSKWNSLVIEYNQIKKWDKESESRSDFYWSLGCERRKEFGLPENFDDELFKAIDDYMWSQKEQLDTDPDTDLQKADLLDVIANLERYVEENHQTCCTKEKPQTIPAEEELHEIQVKEKPQKRLRKEKPQIGNGDEKPKIYSGRKKMPSTEDMEQMMVEKLSENAEMIQAVVNGNLPEMADLEAADSNNIEGFKTDLIRSQGDKLIACLENIVNTMRQFPWLLQDATIASMTGIIGHKTAPSPSGLSTFSLFKALNFHMYCTGLNDNLWFRRPAPYPLGHGV

>Solanum lycopersicum ALP2 XP_004235021 MLCSISTQKSAGSNWLDRLRSSKGFSFADNRNLEQFLTHQTPNGSDSLPSSTETEIRDSNNKDNTGSESSSDPIRPVNESVLPRDQAPAASHNSGDNEELCSVVTNVLSDLFCMGESTSFPKLSVKRGSRKQTNPRFCASSEINGDAVVEGGQRKEETESLDKCRVEIKDSQVKLLEEGHNLNLAEEEDKSNANLMGFSRTEVMVIDTSCAPWKFEKLLFRKKNVWKVRDKKSKTLNLGKKKRKVDVTSEDARGEKKRKFISGHNGYAEKGRECKSSVSEKLQLDDKLEGTCKRTSDSFGQASKKKQRYLKLKKASSSVVLIKSIPTSKKNGVGFAKNSLKPSHR

>Solanum lycopersicum HDP2 XP_004245311 MERSGGSLRTRSQAAPDWTLHESVTLVNEMKATQIECGNSLASFQKWQSTVHNCNSLGVNRSLNQCKRRWESMLEQYNKVKPWESAYWDSFDEERKRELELPEQFDFELFNAIARYLSLEGEDGGGAETDPDTDPEAQQVQGNNAFLEIGPKRQRRRTKTKRYKIEERLNPWRRILNENRKYEQSKMGIKHEASIDAGLEAPRHENSSLEIKRETSSPEEMTELPNLSMVNKVKAEQFHVDNPEELMAATLRENAEMITAITEGNTMDDRDCSLAGLNNFDAGRLHLIRSQGNQLIDCLGKISDTLIQLCDAIHKK

>Vitis vinifera ALP2 XP_004245311 MLCSISTGKSGSKWLDRLRSAKGFPTGNDDDLEHFLTHRDPNLSNSPITKPSDPKSISDSTCSDEKPVQDRSQPPETGEKEWFGIMSNVLAELFNMGDSNQIPKLSGKKSSRKQTNPKICLLSSVRQEDEVPATAPSSGDNSLTEMKDSNGEVKTVNQGKVDCLDAEEEKCNQDLSAYSRSEVTVIDTSCAVWKFEKLLFRKKNVWKVRDKKGKSRSIGRKKRKASECDEQLEARKKMKLSVESFKERNEEESAMPSNEEQNPHNAKKEECKETSDGLSQVPKKRSPFSRLPRKSREGGSPVILVRGSKKTGANLPKITLKDTSRRHKA

>Vitis vinifera HDP2 CBI20803 MEQGEGHQSRVSSSRRTRSQLAPDWTINDSLILVNEIAAVEGECLNALSTYQKWKIIAENCTALDVSRTFNQCRRKWDSLLFEYNKIKKWESRSRNVSFWTLESERRRELGLPVDFERELFKAIDDLVSSQEVRSDTDPGTDPEAEDDRLEVIAEYGPKKQKRREMPQKTTSLEEKEQMMVMKLRENADLIDAIVKGNLVDSVDFGLGGSKNRETLQADFKRRQGDKLIACLRDIADTLDQLRDIVQKCG
