# Supplementary material for: The domesticated transposase ALP2 mediates formation of a novel Polycomb protein complex by direct interaction with MSI1, a core subunit of Polycomb Repressive Complex 2 (PRC2)
Source: PLoS Genet. 2020 May 28;16(5):e1008681. doi: 10.1371/journal.pgen.1008681 (PMC7282668; doi:10.1371/journal.pgen.1008681)
Supplement: Attachment — Submitted filename: Response to Referees.pdf [file pgen.1008681.s017.pdf]

DIVISION *of* BIOLOGICAL SCIENCES
Institute of Molecular Plant Sciences

The University of Edinburgh
Daniel Rutherford Building
King's Buildings
Mayfield Road
Edinburgh EH9 3JH

Fax: 0131 650 5392
Phone: 0131 650 7032
email: Justin.Goodrich@ed.ac.uk
Professor in Developmental Biology
30 Jan 2020

Dear Ortrun,

I am pleased to submit a revised version of our manuscript about the ALP2 domesticated transposase and its role in the formation of a novel Polycomb group protein complex. We are grateful to the referees for helpful and constructive comments and generally positive evaluation. We have performed additional experiments and rewritten parts of the manuscript to address the issues raised. We believe the manuscript is improved as a result and is of broad interest for PLoS Genetics. Our response to the points raised is listed below.

Referee 1

1. The overall function of the ALP proteins was never detected in wild type or single mutant alp plants, but could only be seen in an already quite severe lhp chromatin mutant background (although the single alp2 mutant has a phenotype – Figure 1A). Therefore, I think the authors need to reduce their claims of ALP protein function, because they don't really know this role in normal plants (if they have any role at all). I suggest softening the overall conclusions regarding ALP protein function, including in the abstract and title of the manuscript.

Our IP-MS experiments were all done in wild type backgrounds, so the association of ALPs with the PRC2 occurs in a normal (non PcG compromised) background. We previously (Liang et al, PLoS Genetics 2015) did RNA seq comparisons of wild type vs *alp1* mutants as well as genetic tests in weak *leafy* mutant backgrounds both of which suggested a role for *ALP1* in activating expression of floral homeotic genes. To extend this, we have added an additional experiment (S1 Fig) analyzing the expression of floral homeotic genes following photoperiodic induction in plants shifted from short days to long days and we find a strong effect of *alp1* and *alp2* single mutants. We also changed the title and abstract of the manuscript, as well as the discussion, to soften the overall conclusions as suggested.

1a. The obvious experiment missing here is H3K27me ChIP-seq in wt and the ALP mutants. This experiment would define what role the ALP proteins have in a non-lhp mutant chromatin context.

We fully agree that these are important experiments but feel that they are outside the scope of this manuscript which already contains a substantial amount of novel data to do with the isolation, evolution and proteomics of the ALP-PRC2. We are undertaking H3K27me3 ChIP seq, RNA-seq and biochemical assays to gain more insight in to ALP function but this analysis will be the subject of a separate, more

mechanistic study. We have performed further H3K27me3 ChIP-qPCR at selected targets (revised Fig 8) and do see an increase in H3K27me3 in *clf alp* double mutants relative to *clf* single mutants for some loci, consistent with the partial suppression of the clf mutant phenotype.

2. The introduction reads too long, and I don't think the example of SPM and MU elements that have their methylation changed by the presence of the transposase is a good example of opposing host silencing. In this case, methylation is likely responding to the binding of the transposase protein, and not a direct interference of a host silencing mechanism. There are other examples of direct repression of host silencing that would be more suitable, especially from viruses

We accept that the mechanisms by which several transposases inhibit DNA methylation at specific TE is not well resolved. We removed the paragraph discussing this from the introduction, also reducing length, and we now briefly refer to viruses.

3. HP1 is a very well-studied protein in animals. The authors need to add a sentence or two to explain to the reader how LHP1 is different in plants (does not bind H3K9me).

We added a sentence to clarify this in our introduction.

4. The ALP1-ALP2 proteins interact, and the interaction domain was mapped in Figure 5. Is this domain necessary for interaction that same domain responsible for Harbinger TE nuclease/DNA binding protein interaction? Is this the interaction that the host has reused / domesticated, even though the DNA binding is gone?

We performed additional yeast two hybrid assays (S8 and S9 Fig) using the nuclease and Myb DNA binding proteins encoded by *Ping* transposons (the plant TE category most closely related ALPs and *Harbinger*). We find that the *Ping* proteins interact via the N-terminus of the nuclease and the C-terminus of the Myb, similar to what has been found for Zebrafish *Harbinger* or the Arabidopsis domesticated *Harbinger* proteins HDP1 and HDP2. This suggests that the interaction between the two components has been conserved during domestication in all the cases examined (Harb1/Naif, HDP1/HDP2, ALP1/ALP2). However, the ALP proteins did not interact with the *Ping* proteins in yeast so we think that the domesticated forms have diverged too greatly from the ancestral proteins to recognize them.

4a. What aspects of Harbinger transposons do the authors think lead to their repeated domestication? It seemsfrom their data that it may be the protein-protein interaction. I ask the authors to please speculate on this point in the Discussion section.

There are several distinctive features of Harbinger class TE in plants, namely the modular structure of the transposase complex and the propensity to insert in gene rich regions of the genome. We speculate on this in the final part of the Discussion section.

Minor points

1. The authors define "domestication" as a process whereby the transposase proteins encoded by a TE acquire novel host functions. However, can't domestication function on other TE proteins, such as GAG, Pol, etc…? I favor a more inclusive definition whereby domestication refers to any reuse of a TE for host function, including TE DNA sequences as binding sites and enhancer elements.

We altered the introduction to broaden the definition as requested.

The introduction calls Arabidopsis TEs inactive, but I think this is a misnomer. They are inactive in wt somatic tissues of the reference strain, but active in the species (see eLife 2016;5:e15716).

We changed the sentence in the introduction to remove the word inactive.

2. The first section of the results is very long. I suggest reducing the verboseness, and creating a new paragraph at "To characterize these phenotypes further…"

We shortened the account of the genetics and we broke into paragraphs as requested

3. I did not understand why Figure 2B was important, or where it was referred to in the Results section. This could be moved to the Supplementary data or discussed more in the main text.

We cite Fig 2B in the results now. It is relevant in showing that CLF, ALP1 and ALP2 have overlapping expression, consistent with their acting together in a common complex.

4. Figure 2A is really important to the manuscript. What is the control gene for the qRT-PCR that was used?

We used *Elongation Factor 4A1* (*EiF4A1, At3G13920*), which is now made clear in the legend to Fig 2. *EiF4A1* is not a target of H3K27me3 methylation (e.g. Zhang et al PLoS Biology 2007) and its expression was not altered in our RNA seq comparisons of wild type versus *clf* or wild type versus *alp1* mutants (Liang et al PLoS Genetics 2015).

Referee 2

1. Arguments without supporting evidence should be removed. For example, "as we had independently identified this protein in a yeast two hybrid screen for ALP1 interactors (data not shown)"

We added the results from our yeast two hybrid screen using ALP1 as a bait (S1 dataset)

2. One of the key data in this study is that ALP2 acts as a bridging protein for ALP1 and PRC2 core. Although the authors presented BiFC and IP-MS data supporting this, I would like to suggest that authors perform additional independent experiments to strengthen the argument. E.g. co-IP shown as western blot.

In addition to BiFC and yeast two hybrid data, we performed a pull down from insect cells expressing MSI1 and ALP1 or ALP2 to show that ALP2 but not ALP1 could pull down MSI1 (Fig 7). co-IP is not straightforward as the antibody to Arabidopsis MSI1 we used previously (Liang et al, PLoS Genetics 2015) is no longer available. As an independent experiment we performed 3 replicate experiment using tandem affinity purification to isolate ALP2 complexes from transgenic Arabidopsis suspension culture cells. We found that ALP2 reproducibly co-purifies with MSI1 (S5 Fig, S2 Table). In addition, MSI1 was the most highly enriched protein (other than ALP2 itself) relative to controls in both GFP-ALP2 and GS^{rhino}-ALP2 IP or TAP purifications.

3. Relating the ChIP analyses in Figure 8, it would be nice if ALP1 and ALP2 binding to the target is shown. Plus, I believe ChIP-seq can be carried out with no big difficulties.

Unfortunately we have not been successful in localizing ALP1 or ALP2 in ChIP PCR or ChIP seq experiments despite various attempts with different fixation procedures. It is possible that they do not bind DNA or are associated transiently which prevents us from localizing them with conventional ChIP.

4. A schematic model illustrating the work would be good to be included.

We included a schematic model (Fig 9).

With best wishes

W.J. Goodrich

Justin Goodrich
